# A Novel Technique for Region and Linguistic Specific nTMS-based DTI Fiber Tracking of Language Pathways in Brain Tumor Patients

**DOI:** 10.3389/fnins.2016.00552

**Published:** 2016-12-02

**Authors:** Giovanni Raffa, Ina Bährend, Heike Schneider, Katharina Faust, Antonino Germanò, Peter Vajkoczy, Thomas Picht

**Affiliations:** ^1^Department of Clinical and Experimental Medicine, University of MessinaMessina, Italy; ^2^Neurosurgical Clinic, Department of Neuroscience, University of MessinaMessina, Italy; ^3^Department of Neurosurgery, Charité Universitätsmedizin BerlinBerlin, Germany; ^4^Cluster of Excellence: “Image Knowledge Gestaltung: An Interdisciplinary Laboratory”, Humboldt UniversityBerlin, Germany

**Keywords:** brain tumors, DTI fiber tracking, eloquent areas, language pathways, nTMS, preoperative planning

## Abstract

Navigated transcranial magnetic stimulation (nTMS) has recently been introduced as a non-invasive tool for functional mapping of cortical language areas prior to surgery. It correlates well with intraoperative neurophysiological monitoring (IONM) findings, allowing defining the best surgical strategy to preserve cortical language areas during surgery for language-eloquent tumors. Nevertheless, nTMS allows only for cortical mapping and postoperative language deficits are often caused by injury to subcortical language pathways. Nowadays, the only way to preoperatively visualize language subcortical white matter tracts consists in DTI fiber tracking (DTI-FT). However, standard DTI-FT is based on anatomical landmarks that vary interindividually and can be obscured by the presence of the tumor itself. It has been demonstrated that combining nTMS with DTI-FT allows for a more reliable visualization of the motor pathway in brain tumor patients. Nevertheless, no description about such a combination has been reported for the language network. The aim of the present study is to describe and assess the feasibility and reliability of using cortical seeding areas defined by error type-specific nTMS language mapping (nTMS-positive spots) to perform DTI-FT in patients affected by language-eloquent brain tumors. We describe a novel technique for a nTMS-based DTI-FT to visualize the complex cortico-subcortical connections of the language network. We analyzed quantitative findings, such as fractional anisotropy values and ratios, and the number of visualized connections of nTMS-positive spots with subcortical pathways, and we compared them with results obtained by using the standard DTI-FT technique. We also analyzed the functional concordance between connected cortical nTMS-positive spots and subcortical pathways, and the likelihood of connection for nTMS-positive vs. nTMS-negative cortical spots. We demonstrated, that the nTMS-based approach, especially what we call the “single-spot” strategy, is able to provide a reliable and more detailed reconstruction of the complex cortico-subcortical language network as compared to the standard DTI-FT. We believe this technique represents a beneficial new strategy for customized preoperative planning in patients affected by tumors in presumed language eloquent location, providing anatomo-functional information to plan language-preserving surgery.

## Introduction

Language is the result of the meaningful integration of complex parallel and sequential neuronal processing. The traditional view based on the assumption that Broca's and Wernicke's areas were the unique cortical areas, respectively, involved in the control of articulatory and semantic aspect of speech has been widely overpassed and replaced by a more complex interpretation based on the demonstration of a complex cortico-subcortical neuronal network plastically connecting different brain areas in the dominant perisylvian region, and sometimes also in the non-dominant hemisphere (Chang et al., 2015). Recently, Hickock and Poeppel proposed a plastic “dual stream model” composed by a ventral or “semantic” route and a dorsal or “phonological” route (Hickok and Poeppel, 2004). Several studies documented that different subcortical white matter fiber bundles are involved in the two language routes (Catani et al., 2005; Makris et al., 2005; Duffau et al., 2008a; Glasser and Rilling, 2008; Menjot de Champfleur et al., 2013; Chang et al., 2015). Arcuate fasciculus (AF) is the main component of the dorsal stream, and therefore its disruption is associated with a predominant articulatory aphasia (Catani et al., 2005; Makris et al., 2005; Saur et al., 2008). Conversely, the ventral route is composed of a complex network of white matter fiber bundles, mainly consisting of the inferior longitudinal fasciculus (ILF), the inferior fronto-occipital fasciculus (IFOF) and the uncinate fasciculus (UF). These fiber bundles connect the frontal, temporal and occipital cortices (Papagno, 2011; Axer et al., 2013; Menjot de Champfleur et al., 2013; Chan-Seng et al., 2014; Motomura et al., 2014; Chang et al., 2015).

Actually the standard of care for surgical resection of brain tumors located in proximity of supposed language-eloquent areas is still awake craniotomy with intraoperative electrical stimulation mapping of the complex cortico-subcortical language network (Bello et al., 2007; Duffau et al., 2008b; Sanai et al., 2008; Chang et al., 2015). In the last few years, navigated transcranial magnetic stimulation (nTMS) has emerged as a new method to support such a challenging surgery thanks to its ability to probe the language system non-invasively, based on the induction of virtual lesions even presurgically (Pascual-Leone et al., 1999, 2000; Lioumis et al., 2012; Rogic et al., 2014; Rösler et al., 2014; Sollmann et al., 2015b). It provides a detailed functional map of perisylvian language areas correlating well with intraoperative neurophysiological monitoring (IONM) findings that can be used preoperatively to plan the best customized surgical strategy (Picht et al., 2013; Tarapore et al., 2013). However, nTMS alone only allows for a “cortical” mapping without providing any information about subcortical pathways. Conversely, white matter subcortical tracts can be successfully visualized through DTI fiber tracking (DTI-FT). Hence, in order to overcome the aforementioned limitation, several studies reported the possibility to combine nTMS with DTI-FT for an improved visualization of complex cortico-subcortical brain networks. This strategy has been demonstrated to be more accurate as compared to the standard atlas-based tracking for the motor pathway (Frey et al., 2012; Conti et al., 2014; Rizzo et al., 2014; Weiss et al., 2015). Consequently, theoretically even the use of cortical nTMS language mapping as a “guide” for DTI-FT could improve the visualization of subcortical language fascicles as compared to the standard DTI tracking.

The aim of the present study is to describe a new technique for DTI-FT of subcortical language-involved white matter tracts based on nTMS and to assess its feasibility and reliability as a new tool for a preoperative non-invasive mapping of the complex cortico-subcortical language network prior to surgery of brain tumors in presumed language-eloquent location.

## Materials and methods

### Patients and study design

The study was designed as a prospective case collection study. During 2014 patients scheduled for tumor removal in the vicinity of the presumed essential language areas at the Department of Neurosurgery of the Charité Universitatsmedizin Berlin were screened for enrolment. The inclusion criteria were: (1) existence of a brain tumor compressing/infiltrating areas presumably controlling speech production and comprehension and (2) obscured anatomy of language cortical perisylvian areas and/or language subcortical white matter tracts (AF, IFOF, ILF, UF) due to the mass effect of the tumor or infiltrating growth. The exclusion criteria were: (1) frequent seizures (more than 1/week), (2) existence of a pacemaker or “deep brain stimulation” electrodes and (3) a clinical condition of severe aphasia hampering the ability to correctly perform the object-naming task needed for nTMS mapping. Preoperative aphasia grading of all patients was assessed and adapted to the Aachener Aphasia Test (Huber et al., 1980). A three-level score (0 = no aphasia, 1 = mild aphasia, 2 = moderate aphasia, 3 = severe aphasia) was used. Patients meeting these criteria underwent preoperative magnetic resonance imaging including navigational and diffusion weighted sequences. Subsequently, functional testing of cortical language representation and special anatomical imaging was performed. Ten patients were included in the study. This study was carried out in accordance with the recommendations of the Ethics Commission of the Charité University Hospital (reference # EA4/007/06). All subjects gave written informed consent in accordance with the Declaration of Helsinki.

### MRI acquisition

All patients underwent preoperative 3 Tesla MRI scans for acquisition of T1-weighted 3D MPR, T2-weighted, FLAIR, and DTI sequences (echo train length: 48, TE: 83 ms, TR: 6.800, slice thickness: 3 mm, 20 directions, *b* = 1000). The T1-weighted sequences (echo train length: 1, TE: 2.67 ms, TR: 2.000, matrix size: 256 × 246, slice thickness: 1 mm) were usually used as reference anatomical exam. In case of non-contrast enhancing lesions (i.e., low grade gliomas), T2-weighted (TR/TE 5200 ms/100 ms) fast spin-echo sequences, or T2-weighted inversion recovery (TR/TE/TI 6000 ms/ 150 ms/ 2000 ms) fast spin echo sequence were used.

### nTMS language mapping

All patients underwent repetitive nTMS for localization of language-eloquent cortex. Mapping of both hemispheres was performed by using the Nexstim NBS 4.3 with a NexSpeech module (Nexstim Oy, Helsinki, Finland) with particular regard to the peritumoral area and, in general, to the perisylvian region of the dominant hemisphere. Cranial landmarks used for coregistration between the patient's scalp and MRI scan were the crus of the elix bilaterally, the nasion, and 9 more scalp regions identified by the nTMS software. The maximum stereotactic error allowed by the coregistration software was 5 mm. The stimulation procedure was blocked in case of a higher stereotactic error. Stimulation technique and parameters were already previously described (Picht et al., 2013). Briefly, the resting motor threshold (RMT) for the first dorsal interosseus (FDI) was determined in order to identify the cortical excitability threshold. An object-naming task composed of 122 black and white simple objects presented on a LCD screen located in front of the patient was used. Patients underwent a baseline naming task three times in order to eliminate unrecognized or misnamed objects and to reduce false-positive results as much as possible. Pictures were presented to the patient for 1 s at an individualized inter-picture interval (IPI; 2.5–4 s). According to the most recent literature, the repetitive nTMS stimulation was triggered with picture presentation by using an onset delay of 0 ms (Krieg et al., 2014). The stimulation protocol comprised a train of 5 pulses with a 5 Hz frequency and a 100% RMT intensity. In case of negative mapping (no errors), the number of pulses in a train, frequency and intensity were progressively increased up to 7 pulses, 10 Hz and 120% of RMT, according to the previously described protocol (Picht et al., 2013). Patients were asked to express eventual discomfort during the mapping through the Visual Analogue Scale (VAS) from zero (no pain) to 10 (maximum pain). Stimulation intensity was reduced if patients complained about discomfort during the procedure. Stimulation was performed through a figure-of-eight ventilated coil (maximum diameter of 16.5 cm; minimum diameter of 9 cm) in order to reduce overheating during mapping. It was randomly moved in about 10-mm steps over the peritumoral and perisylvian cortex. The coil was placed perpendicular to the sulcus posterior to the stimulated point to achieve maximum field induction (no offset between the targeted and the stimulated point). During the mapping procedure 80 to 120 sites of the frontal, temporal, and parietal cortex were stimulated 3 times each. The software automatically marked each single spot after stimulation. The same site was stimulated three times without moving the coil and by visual inspection of a correspondence between the coil position and the previous marked spot. Each single site was considered “positive” if the stimulation disrupted object-naming performance at least in two of three stimulations. The average total time for nTMS mapping was about 45 min. The whole procedure was recorded and used for the off-line analysis. The anatomic definition of stimulated areas and the error categories were defined according to Corina et al. (2005, 2010). In particular, speech errors were classified in (1) no-response errors, (2) performance errors, (3) hesitations, (4) semantic paraphasias, (5) phonologic paraphasias. A single site was considered positive for language if a naming error was recorded during stimulation. At the end of the analysis the nTMS map including all the nTMS-positive spots was exported in DICOM format and imported into the surgical planning software. Moreover, three nTMS-negative spots (not able to evoke an error response) from different cortical areas located near the tumor were also exported for each patient in order to analyze their potential connections with language fascicles.

### DTI–3 different approaches

According to the literature, we performed fiber tracking of the most important language-involved subcortical white matter fascicles: AF, ILF, IFOF and UF (Axer et al., 2013; Menjot de Champfleur et al., 2013; Chang et al., 2015). DTI-FT was computed according to three strategies: (1) an atlas-based approach by using traditional anatomical landmarks as seeding regions of interest or ROIs (standard technique); (2) a nTMS-based approach by simultaneously using all the nTMS cortical spots as second seeding ROI (“all-spots” strategy); (3) a nTMS-based approach by using each single nTMS spot sequentially as second seeding ROI (“single-spot” strategy). The average total time was about 20 min for each different DTI-FT procedure.

In a first step, we performed DTI-FT of language pathways by using the standard and the “all-spots” strategy. As second step, we performed DTI-FT of the aforementioned language fascicles by using the nTMS “single-spot” strategy. During this step, we used the same FA stop value to compute the “single-spot” fibers in order to make a reliable comparison between the two nTMS-based techniques. We therefore compared the mean stop value of fractional anisotropy (FA) and the mean FA ratio (defined as FA stop value/FA threshold) required for the best visualization of each fascicle by using the standard vs. the nTMS approach in general (regardless the use of the “single- or all-spots” strategy).

### DTI-FT of language fascicles

The workflow for DTI-FT of language fascicles was performed by using the server-based planning software iPLAN NET 3.0 (Brainlab AG, Feldkirchen, Germany) through a deterministic algorithm. DTI sequences were co-registered with the reference anatomical exam and with the DICOM images containing the nTMS mapping result. Coregistration consisted in a fully automated process performed by the neuronavigation software in order to increase the accuracy of image fusion. After correction for eddy currents, the software computed the FA, Apparent Diffusion Coefficient (ADC) and Directionally Encoded Color (DEC) maps. The DEC map was used for choosing the seeding ROIs for DTI-FT. Tracking was performed by using three different approaches: a standard atlas-based, a nTMS-based “all-spots”, and a nTMS-based “single-spot” strategy.

#### Standard-based strategy

Fiber tracking of AF, ILF, IFOF and UF was performed by placing ROIs over well-established anatomical landmarks as described in the literature (Catani et al., 2002; Catani and Thiebaut de Schotten, 2008). A single ROI placed in the subcortical paraventricular white matter underneath the supramarginal/angular gyrus was used for tracking the AF. Conversely, a two ROIs technique was used for ILF, IFOF and UF. A first ROI was placed at the temporal pole and a second ROI at the posterior portion of the occipital lobe for ILF computation. A first ROI at the posterior part of the occipital lobe and a second ROI around white matter of extreme/external capsule for IFOF. A first ROI at the temporal pole and a second at first slice of the frontal lobe in the coronal view, where temporal and frontal lobe separate, around white matter of extreme/external capsule, for UF.

The FA value to stop tracking was qualitatively chosen in order to obtain the best visualization of each fascicle and to reduce aberrant fibers. Then, the FA was increased up to a value at which no fibers could be visualized anymore. The maximum value at which it was still possible to visualize a single fiber was defined as the FA threshold. The ratio between the FA stop value and the FA threshold was defined as FA ratio. It represents a surrogate measure of the tracking accuracy - the higher the FA ratio used for tracking, the lower the possibility to compute aberrant or artificial fibers.

#### nTMS-based all-spots strategy

All main fascicles (AF, ILF, IFOF, UF) computed by using the standard approach were individually converted into a 3D object and used as first seeding ROI for the nTMS-based tracking. All nTMS spots were then segmented and selected all together as second ROI for fiber tracking (4 trackings: AF + all nTMS spots, ILF + all nTMS spots, IFOF + all nTMS spots, UF + all nTMS spots). Again the FA value to stop tracking was set in order to obtain the best visualization of each fascicle, trying to reduce as much as possible the visualization of aberrant or artificial fibers. Once the best FA value was defined, the FA threshold and the FA ratio were calculated as described for the standard atlas-based strategy.

#### nTMS-based single-spot strategy

As already described for the “all-spots” strategy, the 3D object of each fascicle obtained by using the atlas-based approach was used as first seeding ROI. Then, each single nTMS spot was segmented and used individually as second ROI. We repeated the computation for each single nTMS spot, each time using a different spot as second ROI and for all four main fascicles separately. The FA stop value was the same as the one used for the “all-spots” strategy for each of the main fascicles. This strategy was applied to avoid operator-related FA stop values selection bias that could alter the successive comparison of the number of connected spots obtained by using the two nTMS-based techniques. Finally, to further verify our approach, we repeated the same procedure by using three nTMS-negative spots individually selected as second seeding ROI in each patient, to test the assumption that nTMS-negative spots do not, or significantly less often, connect to main language fascicles.

### Data evaluation

We analyzed the ability of the standard atlas-based strategy and of the nTMS-based approach to reliably compute language pathways without including aberrant or artificial fibers by comparing the mean FA stop values and ratios used for each fascicle. Then, we assessed the ability of the three techniques to visualize the complex cortico-subcortical language network by measuring and comparing the number of cortical nTMS-positive spots connected to the subcortical tracts. These connections were considered as true-positive. Each single connection visualized by the nTMS-based DTI-FT was considered not reliable if fibers (1) had a course that was completely different from the normal course of the corresponding fascicle according to its normal anatomy; (2) had a completely different course as compared to the normal anatomy of the corresponding fascicle, that could not be explained by a compression/dislocation/distortion caused by the tumor.

In order to provide a preliminary statistical validation of our true-positive connections, we analyzed the eventual concordance between the presumed function of each fascicle and the prevalent error type of connected positive nTMS spots. Lastly, with the aim to assess the reliability of the DTI-FT reconstruction of the language network, we analyzed the number of connected nTMS-negative spots. These were considered as false-positive connections. According to the potential connection of each single spot (regardless positive or negative) to a maximum of 4 fascicles (AF, IFOF, ILF, UF), we analyzed and compared the likelihood of false-positive subcortical connections with the likelihood of true-positive ones.

### Statistical analysis

Paired Student's *t*-test was used to compare (1) FA values and ratios, (2) the number of cortical nTMS-spots (positive or negative) connected to subcortical tracts obtained by using the three different techniques, (3) the likelihood of connections for positive and negative nTMS spots (true-positive vs. false-positive connections). The ANOVA test was used to analyze the different distribution of error types among connected nTMS-positive spots. Statistical significance was defined as a *p* < 0.05. GraphPad Prism version 6.00 for Windows was used for all the statistical analysis (GraphPad Software, La Jolla, California, USA, www.graphpad.com).

## Results

We collected neuroimaging data of 10 patients (6 males, 4 females; mean age 57 ± 14) suffering from left perisylvian brain tumors (5 glioblastomas, 3 anaplastic astrocytomas, 1 oligoastrocytoma, 1 hemangioma) that underwent preoperative language mapping using repetitive nTMS. Table 1 summarizes patients' characteristics.

**Table 1:** **Patients' salient clinical characteristics**.

**Patients**	**Age**	**Gender**	**Handendness**	**Histology**	**Location**	**Preoperative aphasia score**
#1	53	m	Right handed	Glioblastoma	Left, temporal	1
#2	67	m	Right handed	Anaplastic Astrocytoma	Left, temporo-polar	0
#3	60	m	Right handed	Glioblastoma	Left, supra-opercular	1
#4	51	m	Right handed	Glioblastoma	Left, temporo-parietal	0
#5	77	m	Right handed	Glioblastoma	Left, fronto-opercular	1
#6	77	m	Right handed	Glioblastoma	Left, fronto-opercular	0
#7	40	f	Right handed	Oligoastrocytoma	Left, temporal	0
#8	41	f	Right handed	Anaplastic Astrocytoma	Left, frontal	0
#9	65	f	Right handed	Anaplastic Astrocytoma	Left, fronto-opercular	0/1
#10	40	f	Left handed	Hemangioma	Left, frontal	0

**Figure 1:**
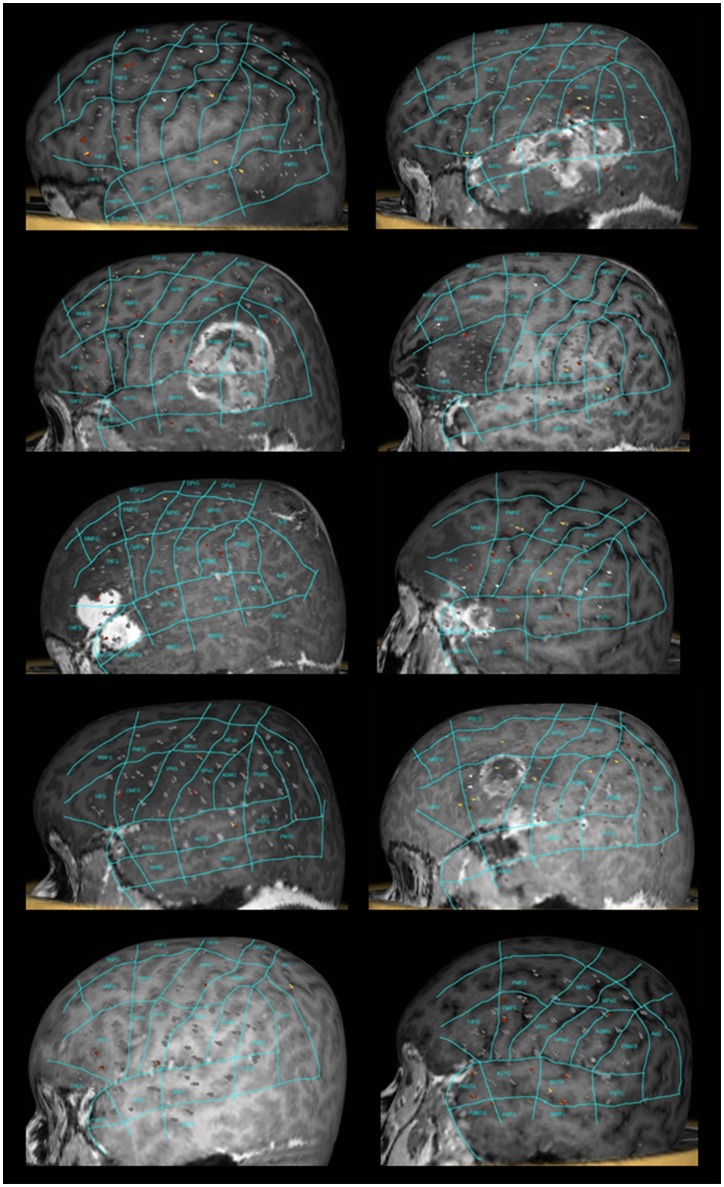
**nTMS language mapping of all patients according to the cortical parcellation system described by Corina et al. (2005, 2010)**. Errors were classified according to a color-coded technique (no response errors: white spots; hesitations, performance and phonological errors: red spots; semantic errors: yellow spots).

All patients underwent nTMS-mapping of the left perisylvian region in order to identify essential language sites (Figure 1). We observed preserved language function in all cases and language mapping was successfully performed in all patients. Baseline errors during preoperative object naming ranged from 2.54 to 52.08% (median 18.38%) of shown objects. The left hemisphere was stimulated at 152 to 211 sites (median 188.5 sites). During stimulation, 6 to 21 naming errors (median 13 errors) were observed. The error rate ranged from 3.04 to 13.81% (median 7.1%). As referred to the map defined by Corina et al. (2005, 2010), errors were recorded more frequently at opercular inferior frontal gyrus (opIFG; 13.6% of total errors), posterior middle frontal gyrus (pMFG; 10.4%), triangular inferior frontal gyrus (trIFG; 9.6%), anterior supramarginal gyrus (aSMG; 9.6%) and ventral precentral gyrus (vPrG; 8%) (Tables 2, 3). The mean VAS score during the stimulation procedure was 5.3 ± 2.7. In all cases, we were able to obtain DTI-FT of AF, ILF, IFOF and UF by using all three techniques.

**Table 2:** **nTMS mapping findings according to Corina's cortical parcellation system (Corina et al., 2005, 2010)**.

	**Case #1**	**Case #2**	**Case #3**	**Case #4**	**Case #5**	**Case #6**	**Case #7**	**Case #8**	**Case #9**	**Case #10**
aSMG	4	1	1	1	0	1	0	0	2	2
aSTG	1	1	0	0	4	0	0	0	0	1
mSTG	1	4	0	0	1	0	1	0	0	0
opIFG	1	4	6	0	2	2	1	0	0	1
poIMTG	0	1	0	0	0	0	0	0	0	0
poISTG	0	3	0	0	0	0	0	0	0	1
anG	1	0	1	1	0	1	1	0	0	0
mPrG	2	0	1	1	1	0	1	1	0	0
pMFG	0	0	3	3	3	0	2	0	1	1
pSMG	0	0	1	0	2	0	0	0	0	0
trIFG	0	0	1	2	2	0	3	2	1	1
vPrG	1	0	2	1	1	2	1	2	0	0
dPrG	0	0	0	1	0	0	0	0	0	0
mMFG	0	0	0	1	0	0	0	0	0	0
mMTG	0	0	0	1	0	2	0	0	0	0
mPoG	1	0	0	2	0	1	0	0	0	0
pSFG	1	0	0	1	0	0	0	0	0	0
vPoG	1	0	0	1	3	1	1	0	0	0
SPL	0	0	0	0	0	0	0	1	0	0
aMFG	0	0	0	0	0	0	0	0	1	0
pSTG	2	0	0	0	1	0	0	0	1	0
aMTG	0	0	0	0	1	0	0	0	0	0
pMTG	1	0	0	0	0	0	1	0	0	0
Total Err	17	14	16	16	21	10	12	6	6	7
Total trials	211	156	170	202	152	186	191	186	197	198
Baseline errors (%)	16.10	11.33	52.08	20.66	38.75	6.66	35.33	14.41	28.00	2.54
Error Rate (%)	8.05	8.97	9.41	7.92	13.81	5.37	6.28	3.22	3.04	3.53

**Table 3:** **Abbreviations of anatomical areas according to Corina's parcellation system (Corina et al., 2005, 2010)**.

**Abbreviation**	**Anatomy**
aMTG	Anterior middle temporal gyrus
anG	Angular gyrus
aSMG	Anterior supramarginal gyrus
aSTG	Anterior superior temporal gyrus
dPoG	Dorsal postcentral gyrus
dPrG	Dorsal precentral gyrus
mMFG	Middle middle frontal gyrus
mMTG	Middle middle temporal gyrus
mPoG	Middle postcentral gyrus
mPrG	Middle precentral gyrus
mSFG	Middle superior frontal gyrus
mSTG	Middle superior temporal gyrus
opIFG	Opercular inferior frontal gyrus
pMFG	Posterior middle frontal gyrus
pMTG	Posterior middle temporal gyrus
pSFG	Posterior superior frontal gyrus
pSMG	Posterior supramarginal gyrus
pSTG	Posterior superior temporal gyrus
SPL	Superior parietal lobe
trIFG	Triangular inferior frontal gyrus
vPoG	Ventral postcentral gyrus
vPrG	Ventral precentral gyrus

### Comparison of FA values and ratio by using the standard vs. the nTMS-based strategy

Mean FA values and ratios were higher when using the nTMS-based approach (regardless of using the “single-spot” or the “all-spots strategy”) as compared to the standard atlas-based technique for each fascicle.

**Figure 2:**
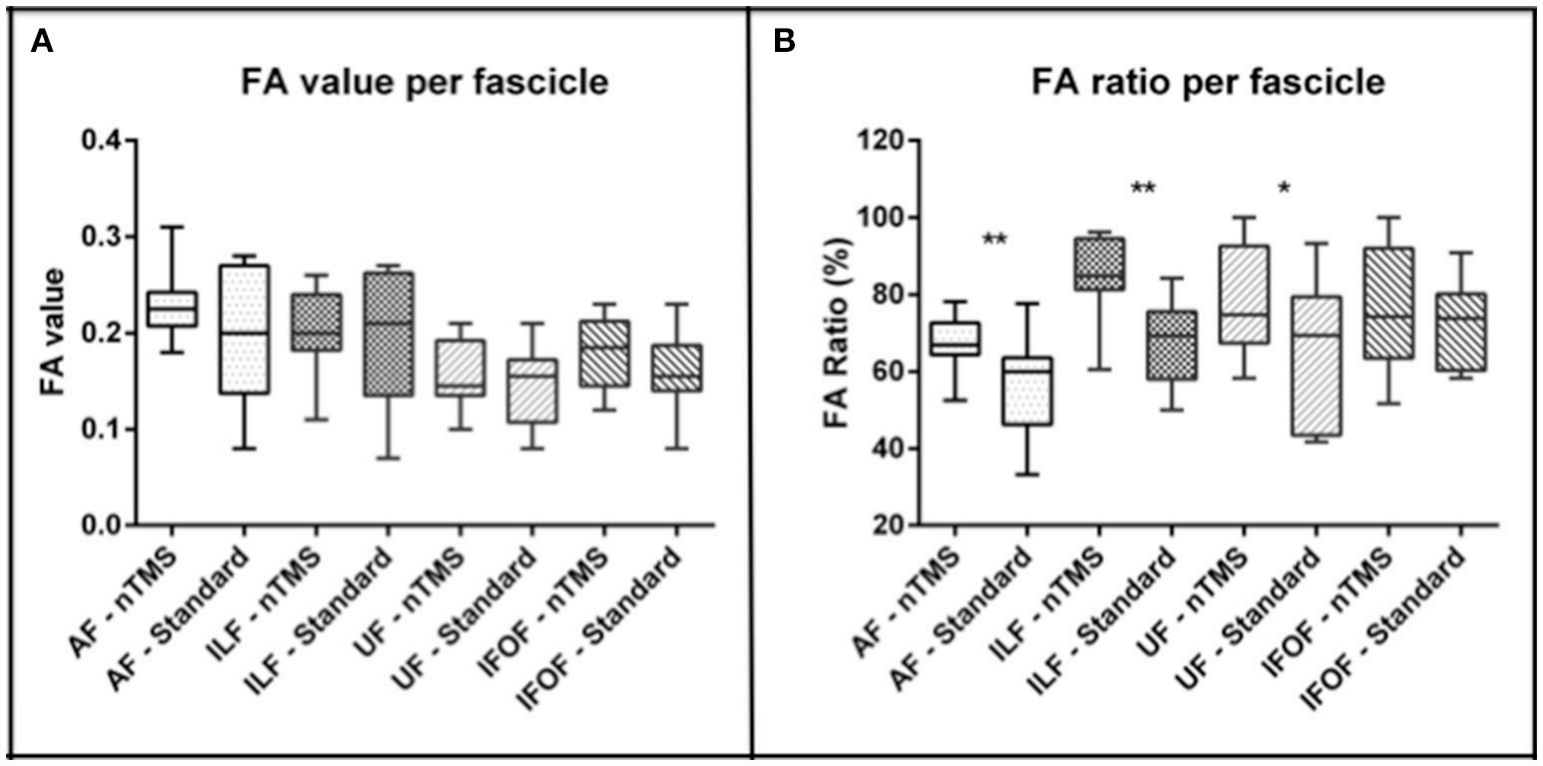
**Analysis of the FA value and ratio of each computed fascicle by using the nTMS strategy vs. the standard atlas-based technique. (A)** A trend toward an increase of FA values was observed by using the nTMS-based technique. **(B)** The FA ratio used for the nTMS-based DTI-FT was higher for each fascicle as compared to the standard technique. The difference was significant for AF (^**^p = 0.006), ILF (^**^p = 0.002), and UF (^*^p = 0.04).

The mean FA stop value was, respectively, 0.22 ± 0.03 vs. 0.19 ± 0.06 for AF, 0.20 ± 0.04 vs. 0.19 ± 0.06 for ILF, 0.17 ± 0.03 vs. 0.15 ± 0.04 for IFOF, and 0.15 ± 0.03 vs. 0.14 ± 0.04 for UF (Figure 2A). These differences between the two techniques were not statistically significant.

**Figure 3:**
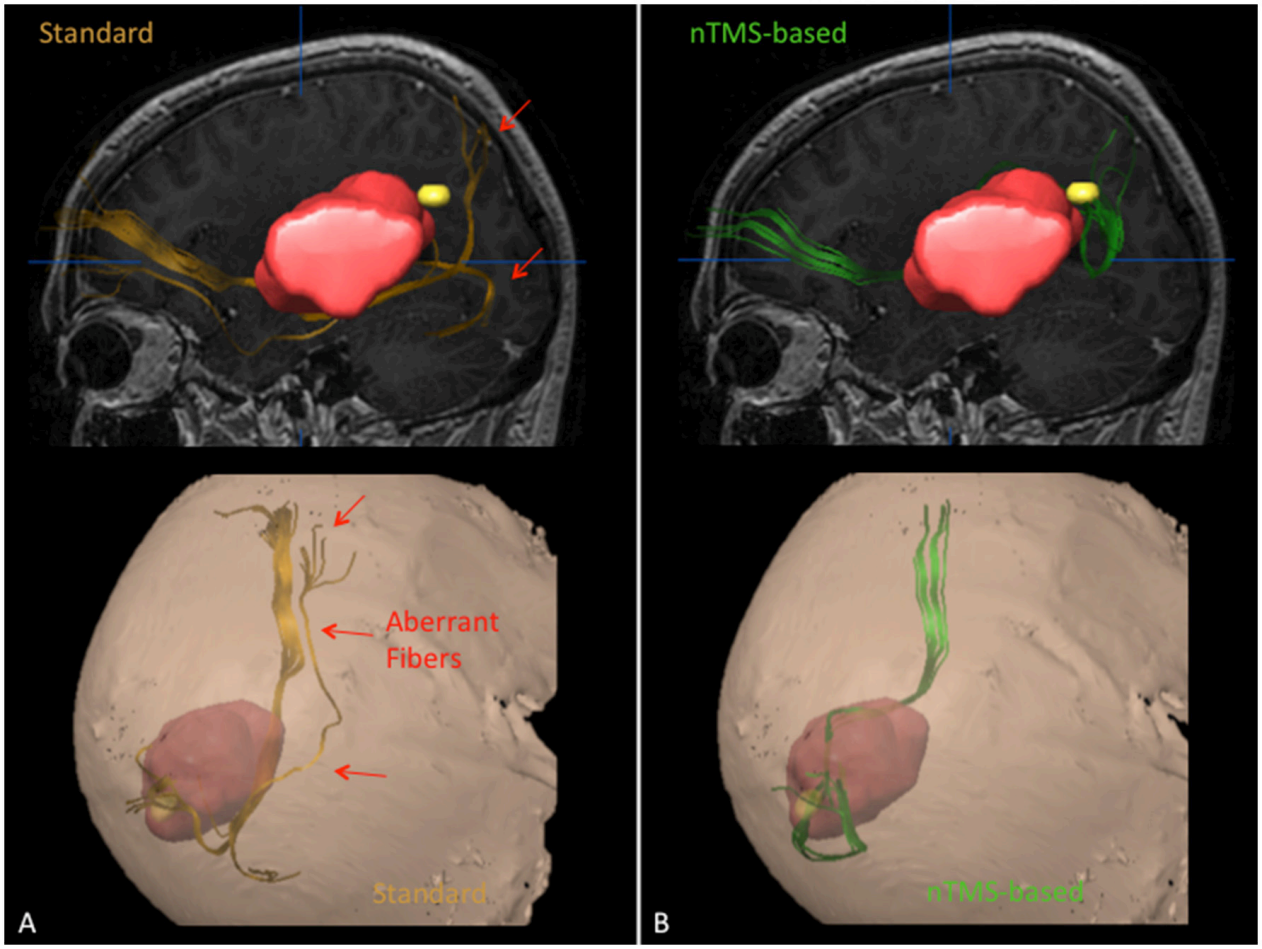
**Case example showing the ability of the nTMS strategy to reduce the visualization of aberrant/false positive fibers as compared to the standard approach for computation of the IFOF in a case of a patient with a large left perisylvian tumor**. The IFOF was computed by using the standard and the nTMS-based DTI-FT, obtaining two completely different results. **(A)** The IFOF computed by using the standard approach (orange) appears running below the lesion. Some aberrant fibers are visualized medially to its main trunk. **(B)** The IFOF obtained by using the nTMS-based strategy (green) is running above the lesion, being displaced upward. No aberrant fibers are visualized anymore.

The mean FA ratio (%) was 67.5 ± 7.2 vs. 55.8 ± 12.8 for AF, 85 ± 10.6 vs. 67.4 ± 10.6 for ILF, 76 ± 15.8 vs. 72.5 ± 10.6 for IFOF, and 77.6 ± 13.6 vs. 66.2 ± 18.1 for UF. The difference was statistically significant for AF, ILF and UF (*p* = 0.006, *p* = 0.002, *p* = 0.04) (Figure 2B). In particular, through the nTMS-based approach we were able to obtain a good representation of the main language fascicles by using a FA ratio ranging from 60% to 75% for the AF, from 74 to 96% for the ILF, form 60 to 92% for the IFOF, and from 64 to 91% for the UF (Figure 3).

### Comparison of cortical spots connected to language fascicles by using all three techniques

**Figure 4:**
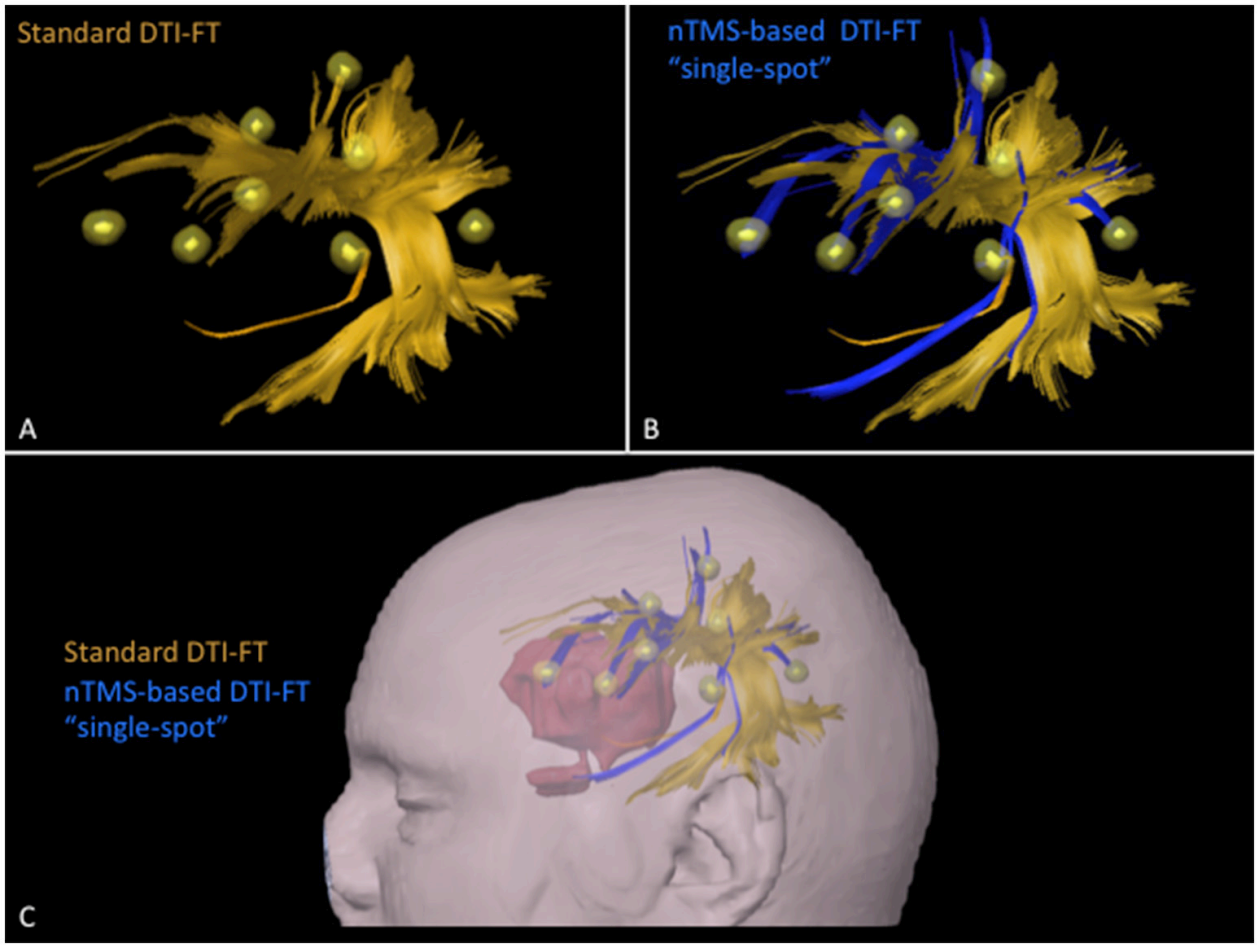
**Case example of a patient affected by a large left frontal tumor**. The AF was computed by using the **(A)** standard and the **(B)** nTMS-based “single spot” DTI-FT. The AF computed by the nTMS strategy shows an evident higher number of connections (blue fibers) to the nTMS spots as compared to the standard technique. **(C)** These new fibers obtained by using the nTMS-based “single spot” strategy appear to run just above the tumor. The visualization of these fibers could provide additional helpful information for the presurgical planning that should be set trying to approach the tumor preserving AF fibers.

The total number of cortical nTMS spots connected to any of the language fascicles was significantly higher by using the nTMS-based “single-spot” strategy as compared to the other two techniques (standard vs. nTMS “all-spots” *p* = 0.002; standard vs. nTMS “single-spot” *p* = 0.0004; nTMS “all-spots” vs. nTMS “single-spot” *p* = 0.009). Figure 4 shows the difference of connected spots obtained by using the nTMS “single spot” strategy as compared to the standard technique, in a case example of a patient affected by a large left frontal tumor in which the AF was computed.

When comparing the number of cortical nTMS spots connected to each single fascicle among the three techniques, we still observed a significantly higher number of connections (true-positive) by using the nTMS-based strategy for all fascicles (Table 4).

**Table 4:** **Comparison of the number of cortical nTMS spots connected to each single fascicle by using the three different DTI-FT techniques**.

**Fascicles**	**Mean ± SD of connected spots (median, range)**			**Comparison – statistical significance**
	***Standard DTI-FT***	***nTMS-based “all-spots” DTI-FT***	***nTMS-based “single-spot” DTI-FT***	
AF	1.9 ± 1.5	4.1 ± 1.4	5 ± 1.7	“all-spots” vs. standard *p = 0.0003*
	(2, 0–5)	(4, 2–6)	(4.5, 3–8)	“single-spot” vs. standard *p* < 0.0001
				“single-spot” vs. “all-spots” *p = 0.01*
UF	0.2 ± 0.4	2.8 ± 1.5	3.4 ± 1.5	“all-spots” vs. standard *p = 0.0003*
	(0, 0–1)	(3, 1–5)	(3, 1–6)	“single-spot” vs. standard *p < 0.0001*
				“single-spot” vs. “all-spots” *p = 0.02*
IFOF	0	3.5 ± 1.6	3.9 ± 1.5	“all-spots” vs. standard *p < 0.0001*
		(3, 2–6)	(3.5, 2–6)	“single-spot” vs. standard *p < 0.0001*
				“single-spot” vs. “all-spots” *p = 0.03*
ILF	0	2.5 ± 1.1	3.1 ± 1.5	“all-spots” vs. standard *p < 0.0001*
		(2.5, 1–5)	(3, 1–6)	“single-spot” vs. standard *p = 0.0001*
				“single-spot” vs. “all-spots” *p = 0.05*

**Figure 5:**
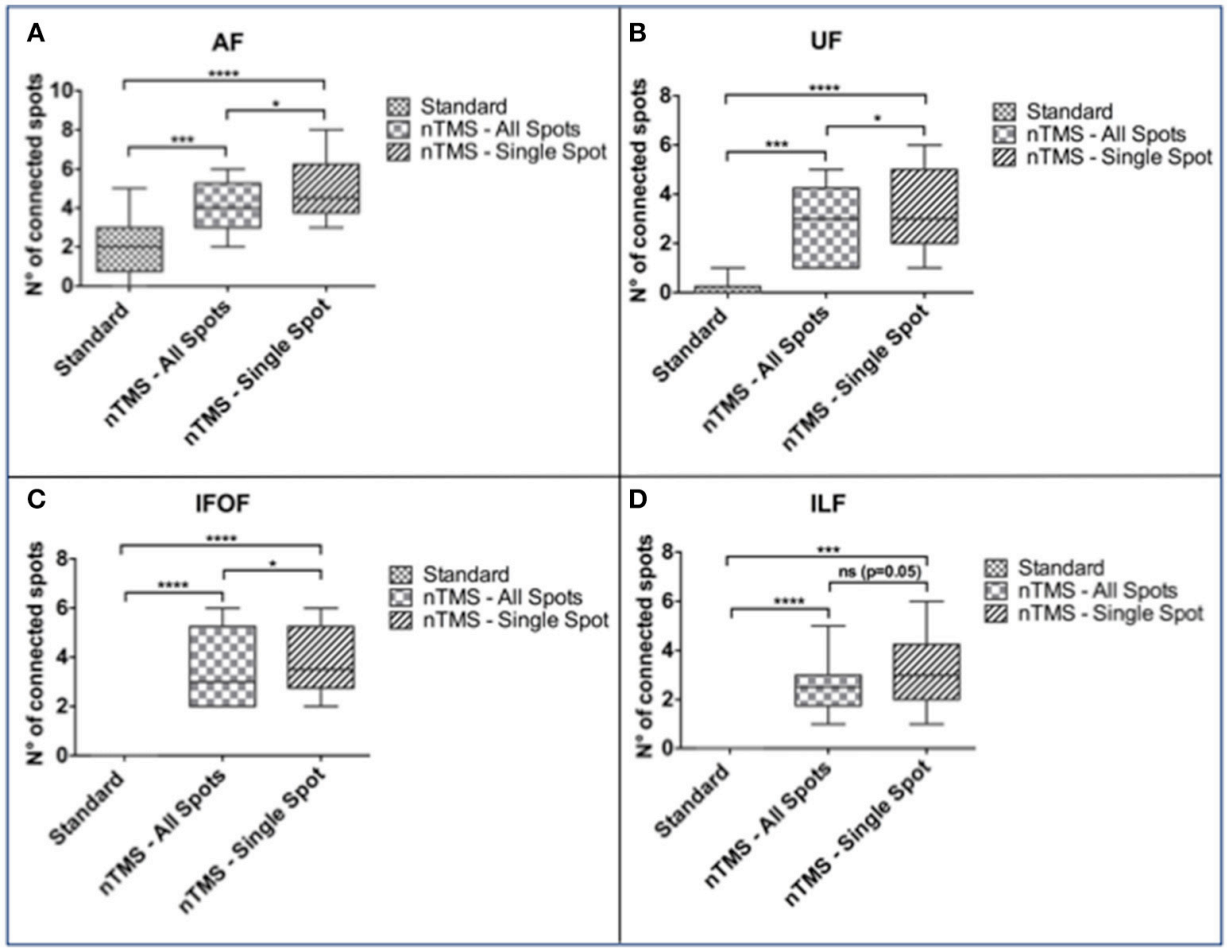
**Comparison of the number of cortical nTMS spots connected to each language fascicle by using the three different DTI-FT techniques**. The “single-spot” nTMS-based strategy showed a significantly higher number of connected nTMS spots as compared to the other two techniques for the **(A)** AF, **(B)** UF, and **(C)** IFOF. **(D)** Regarding ILF, the difference was statistically significant only when comparing the standard vs. the nTMS “all-spots” and “single-spot” strategies, but it was not significant when comparing the two different nTMS-based techniques. ^*^p < 0.05; ^***^p < 0.001; ^****^p < 0.0001.

In particular, the nTMS-based “single-spot” strategy resulted in a significantly higher number of connected spots as compared to the other two techniques for AF, UF, and IFOF (Figure 5).

**Figure 6:**
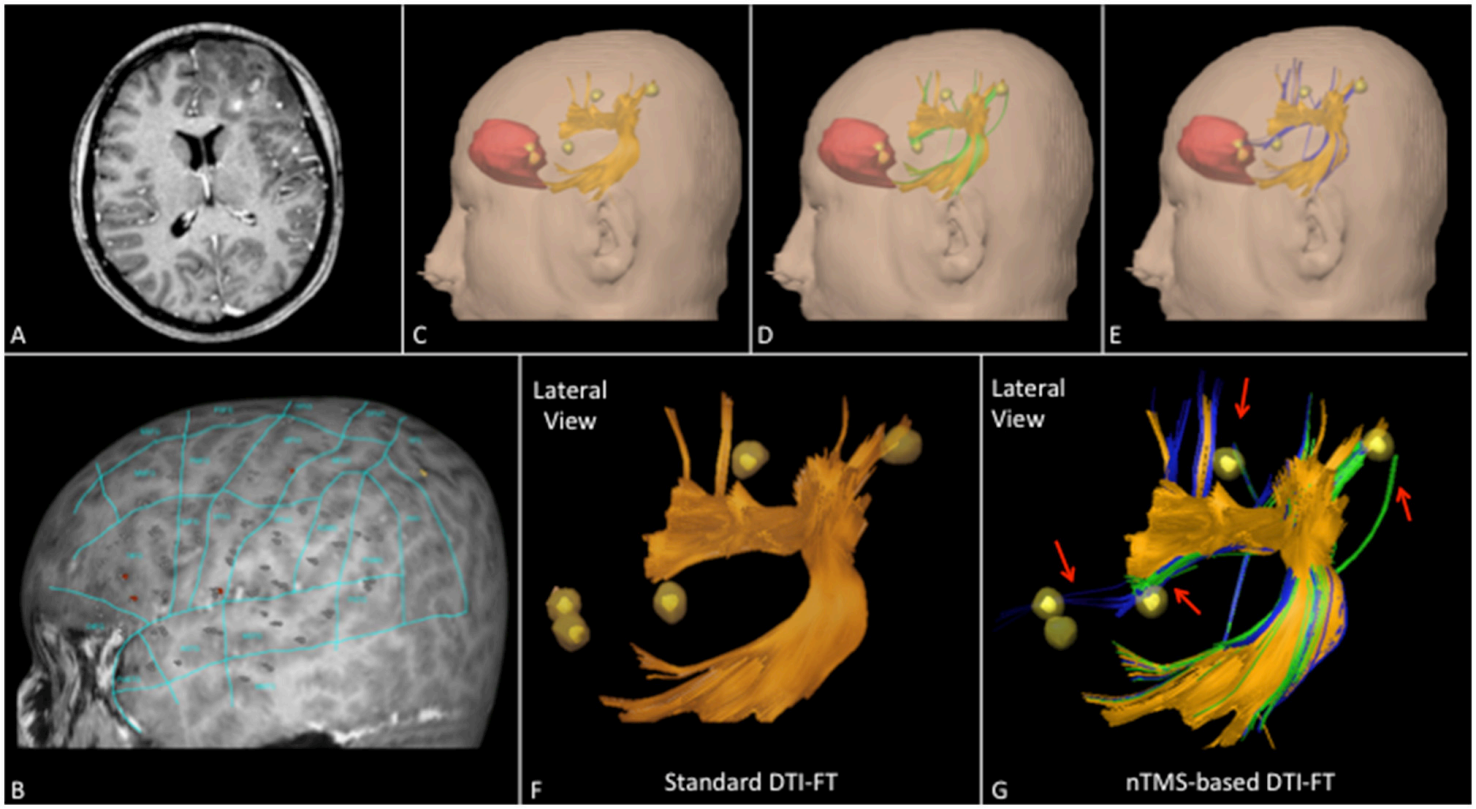
**(A)** T1-weighted contrast enhanced sequence showing a case of a left frontal anaplastic astrocytoma. **(B)** nTMS language mapping findings according to Corina's cortical parcellation system (Corina et al., 2005, 2010). **(C)** Lateral view of AF (orange) computed by using the standard DTI-FT technique showing its spatial relationship with nTMS spots (yellow) and tumor (red). **(D)** Lateral view of AF computed by using the nTMS-based “all-spots”, and **(E)** “single-spot” strategy showing new additional fibers (respectively green and blue) connecting AF to nTMS spots. **(F)** 3D reconstruction of AF by using standard and **(G)** nTMS-based DTI-FT. It's interesting to note the additional fibers obtained by using the “all-spots” and the “single-spot” strategy connecting AF to cortical nTMS spots (red arrows).

**Figure 7:**
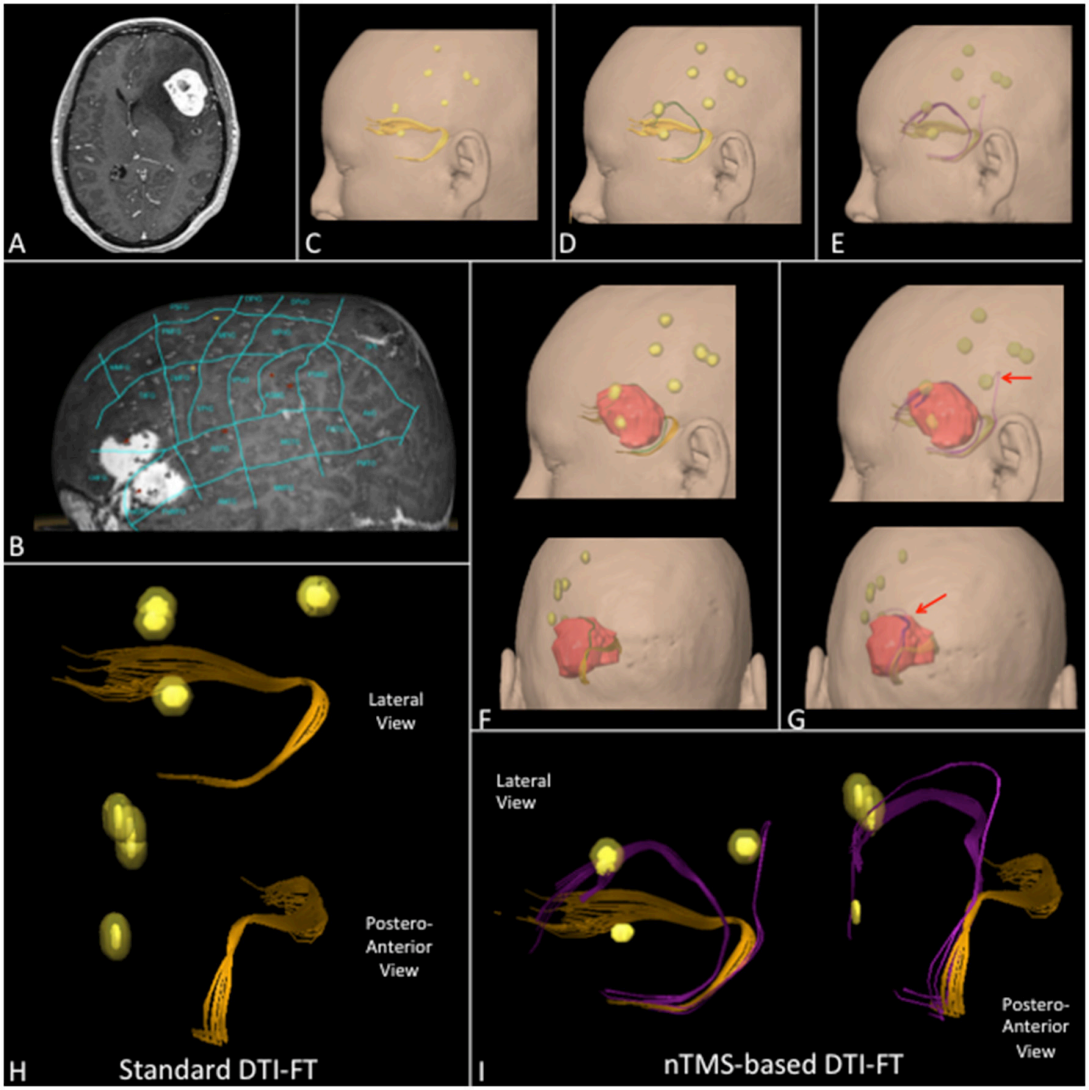
**(A)** T1-weighted contrast enhanced sequence showing a case of a left frontal hemangioma. **(B)** nTMS language mapping findings according to Corina's cortical parcellation system (Corina et al., 2005, 2010). **(C)** Lateral view of UF (orange) computed by using the standard, **(D)** the nTMS-based “all-spots” and **(E)** the “single-spot” DTI-FT showing additional fibers (respectively green and purple). **(F)** Lateral and postero-anterior view of UF by using the “all-spot” and **(G)** the “single-spot” strategy and its spatial relationship with nTMS spots (yellow) and tumor (red). Additional fibers (purple) connecting the UF with nTMS spots obtained by using the “single-spot” technique are indicated by red arrows. **(H)** 3D reconstruction of the UF in the lateral and postero-anterior view computed by using standard and **(I)** nTMS-based “single-spot” DTI FT. Additional fibers (purple) and their connection with nTMS-spots are evident.

**Figure 8:**
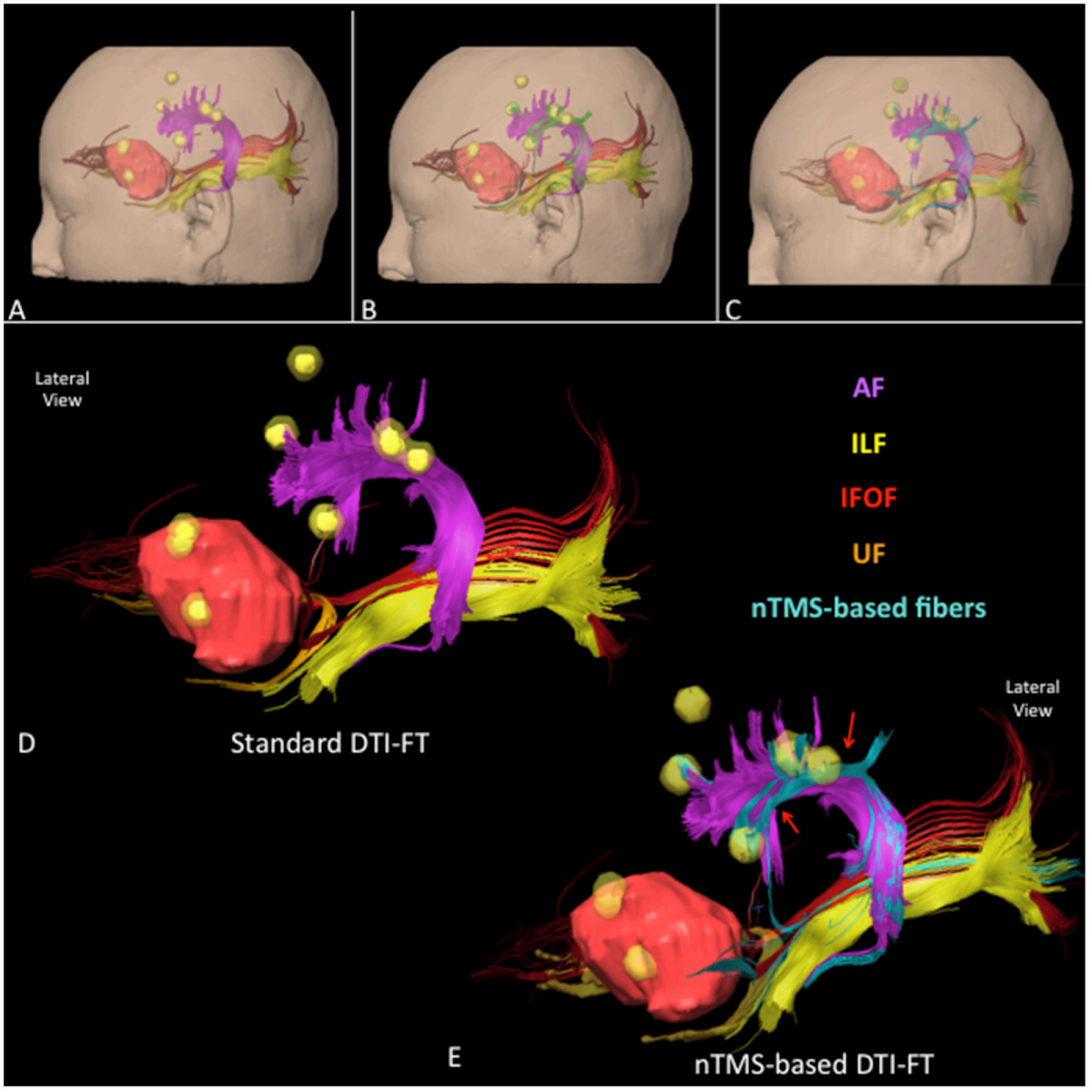
**Lateral view of all language fascicles in a case of a left frontal anaplastic astrocytoma obtained through (A)** standard, **(B)** nTMS-based “all-spots”, and **(C)** “single-spot” DTI-FT. The spatial relationship between AF (purple), UF (orange), IFOF (red), ILF (light yellow), tumor (light red) and nTMS spots (yellow) is showed. Additional fibers are depicted in green for the “all-spot” and in light blue for the “single-spot” strategy. **(D)** 3D reconstruction of the language cortico-subcortical network by using the standard, and **(E)** the nTMS-based “single-spot” DTI-FT. Additional fibers connecting fascicles to cortical nTMS spots are highlighted (light blue fibers, red arrows).

We report some examples of graphical differences between the nTMS-based and the standard atlas-based techniques in selected cases for AF (Figure 6), UF (Figure 7), and collectively for all fascicles (Figure 8).

### Analysis of correlation between fascicles' presumed functions and the error type of connected spots

**Figure 9:**
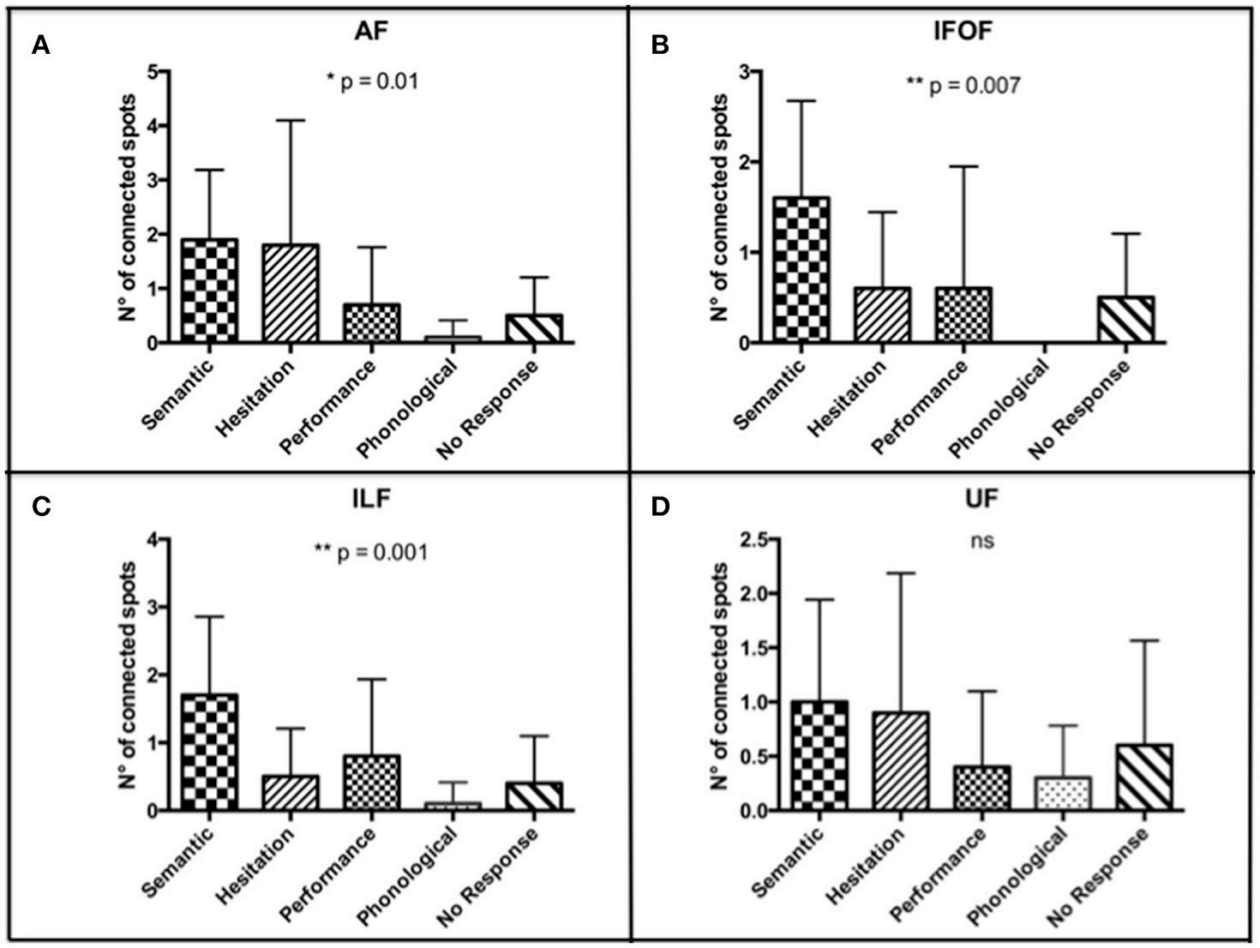
**Distribution of error types among connected spots for each single language fascicle computed by using the nTMS-based “single spot” technique**. The most frequent error types were **(A)** semantic and hesitations for the AF (*p* = 0.01); **(B)** semantic for the IFOF (*p* = 0.007); **(C)** semantic for the ILF (*p* = 0.001); **(D)** semantic, hesitations, and no response for the UF (ns). These data confirmed a good concordance between the expected language function of each fascicle and the error type of the connected cortical nTMS spots.

According to the higher number of connected spots that we found by using the nTMS-based “single-spot” strategy, we analyzed the distribution of error types among connected spots for each single language fascicle computed by using the above-mentioned technique. We observed that the distribution of error types among connected spots was significantly different for the AF (*p* = 0.01), IFOF (*p* = 0.007), and ILF (*p* = 0.001). The distribution was not significantly different for the UF (Figure 9).

### Analysis of the likelihood of true-positive and false-positive connections

**Figure 10:**
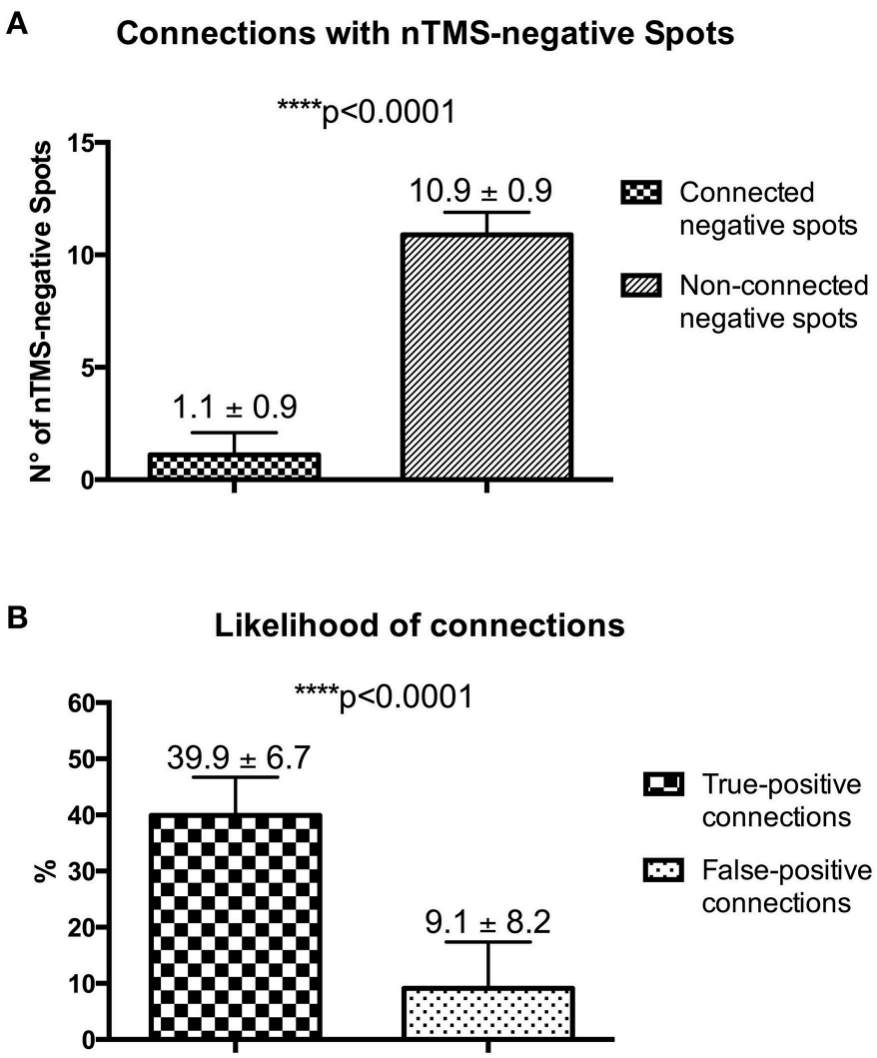
**(A)** Analysis of connections with nTMS-negative spots. **(B)** Difference of the likelihood of true-positive (nTMS-positive spots) and false-positive (nTMS-negative spots) connections.

With the nTMS-DTI-FT “single spot” strategy being able to visualize a higher number of connected positive spots as compared to the other techniques, we used it also to analyze the number of connections with nTMS-negative spots. The mean number of connected negative spots was significantly lower as compared to the non-connected ones [1.1 ± 0.9 (median 1.5, range 0–2) vs. 10.9 ± 0.9 (median 10.5, range 10–12); *p* < 0.0001] (Figure 10A).

According to potential connections of both positive and negative nTMS spots, in our series we observed a likelihood of connections for nTMS-positive spots (true-positive connections) of 39.9 ± 6.7 % (median 38.35, range 31.25–50) vs. a likelihood of connection for nTMS-negative spots (false-positive connections) of 9.1 ± 8.2 % (median 12.45, range 0–16.6) (Figure 10B).

## Discussion

DTI-FT represents the only preoperative technique for visualization of the complex subcortical language network. Different studies have already demonstrated the utility of intraoperative neurophysiological mapping of subcortical language fascicles, especially if guided by DTI-FT, in reducing postoperative speech deficits after surgery of brain tumors located in the dominant perisylvian region (Bello et al., 2008; Leclercq et al., 2010; Chan-Seng et al., 2014). However, standard DTI-FT has several limitations. The most important is represented by a high inter-examiner variability due to the need for priori defined anatomical and functional landmarks for fiber tracking. Brain tumors frequently cause cortical and subcortical structural alterations, either by mass-effect or by inducing functional reorganization (Ius et al., 2011). Therefore, in brain tumor patients anatomical landmarks alone cannot be considered reliable for predicting eloquence, or to identify functional regions to be used as seeding region for an accurate DTI-FT (Pouratian and Bookheimer, 2010). This limitation can be overcome by identifying functional areas through nTMS cortical mapping. It has been demonstrated that the use of nTMS mapping of functional motor cortex as seeding region for corticospinal tract (CST) reconstruction reduces the inter-examiner variability of DTI-FT, providing a more accurate CST visualization as compared to the standard atlas-based technique (Frey et al., 2012; Conti et al., 2014; Weiss et al., 2015). However, to our knowledge, so far the use of this new approach for DTI-FT of language pathways has been reported only in a single case report (Sollmann et al., 2015a). In the present study, we combined the well-established approach to DTI-FT of language subcortical white matter tracts (Catani and Thiebaut de Schotten, 2008), with the unique information provided by cortical nTMS language mapping. The novel aspect of this approach is the ability to focus not only on the subcortical component of the language network but also on the functional organization of cortical language areas. Language subcortical tracts can be reliably identified through DTI-FT by using anatomical landmarks, whereas cortical areas are not predictable unless using functional mapping data. nTMS is a valid technique to accomplish this mapping pre-operatively in a non-invasive way (Picht et al., 2013). In our study, the combination of standard visualization of the main subcortical language tracts with the cortical nTMS information allowed for displaying connections between subcortical tracts and cortical language sites otherwise not accessible to standard DTI analysis. In addition, we demonstrated that cortical connections could be better identified by using the nTMS-based “single-spot” technique as compared to the “all-spot” strategy. Conversely, standard DTI-FT, being based only on anatomical landmarks, resulted in a low ability in displaying this kind of cortical connections, underestimating the underlying language network.

In particular, the most frequent error types among connected spots were semantic and hesitation errors for the AF, semantic for the ILF and the IFOF, and semantic, hesitations, and no response for the UF. These data confirmed a good concordance between the expected language function of each fascicle and the error type of the connected cortical nTMS spots. Table 5 shows the details of errors distribution for each single fascicle.

**Table 5:** **Description of the different distribution of errors among connected spots, obtained by using the nTMS-based “single spot” approach, for each language fascicle**.

**Fascicles**	**Mean ± SD of connected spots (median, range)**					***p-value***
	***Semantic***	***Hesitation***	***Performance***	***Phonological***	***No response***	
AF	1.9 ± 1.28	1.80 ± 2.30	0.70 ± 1.05	0.10 ± 0.31	0.50 ± 1.70	*0.01*
	(2, 0–4)	(0.5, 0–6)	(0, 0–3)	(0, 0–1)	(0, 0–2)	
UF	1 ± 0.94	0.90 ± 1.28	0.40 ± 0.69	0.30 ± 0.48	0.60 ± 0.96	*0.36*
	(1, 0–3)	(0.5, 0–4)	(0, 0–2)	(0, 0–1)	(0, 0–3)	
IFOF	1.60 ± 1.07	0.60 ± 0.84	0.60 ± 1.35	0	0.50 ± 0.70	*0.007*
	(2, 0–3)	(0, 0–2)	(0, 0–4)	0	(0, 0–2)	
ILF	1.70 ± 1.16	0.50 ± 0.70	0.80 ± 1.13	0.10 ± 0.31	0.40 ± 0.69	*0.001*
	(1.5, 0–4)	(0, 0–2)	(0, 0–3)	(0, 0–1)	(0, 0–2)	

Moreover, accuracy of DTI-FT is strictly linked to the FA value used for tracking. FA was described for the first time by Basser and Pierpaoli as a quantitative measure of white matter anisotropy in diffusion tensor MR-imaging (Basser and Pierpaoli, 1996). It reflects the microstructural properties of white matter, measuring the anisotropic movement of water according to axon size, density and orientation (Lawrenz et al., 2015). High FA values indicate high axons size and density, and/or their highly coherent orientation, and vice versa. To run DTI software, it is a mandatory to define the FA value at which the software should stop tracking in the DWI sequences. Briefly, the software continues to track fibers in each voxel of the DTI map unless an FA value lower than the a priori defined FA stop value is found. Therefore, low FA stop values can produce an over-representation of tracts, including aberrant fibers with low FA, depicting potentially a large number of false positive fibers. Conversely, high FA stop values can dramatically reduce the number of represented fibers, increasing the likelihood of false-negative results. In addition, FA values in the DTI map can be modified by different pathological conditions, including brain tumors (Yen et al., 2009; Zolal et al., 2013). For instance, peritumoral edema, reflecting a higher free water content, increases the isotropy of white matter and causes a decrease of FA values (Lu et al., 2003). This hampers the ability to accurately reproduce the anatomy of subcortical tracts that appear underestimated, unless using lower FA stop values. Similar findings have been reported for white matter tracts infiltration or disruption (Yen et al., 2009). Therefore, the choice of the best FA value for tracking must be tailored to the individual case in order to increase the accuracy of DTI. Frey et al. described an elegant method to overcome this main issue in DTI-FT, demonstrating that tracking at 75% of the individual FA threshold (FA ratio = 75%) using an nTMS-based strategy leads to a more reliable representation of CST as compared to the standard DTI-FT (Frey et al., 2012). This strategy, apart from representing a customized approach to standardize DTI-FT and to reduce operator-variability, improves DTI-FT accuracy and results. In the present study, the use of the nTMS-based strategy allowed us to improve DTI-FT reconstruction of language pathways, significantly increasing both the absolute FA stop value and the FA value/FA threshold ratio. This resulted in a better and more reliable definition of all language tracts as compared to the standard atlas-based DTI-FT, reducing the possibility to obtain false-positive fibers. This was demonstrated by the significantly higher values of the FA ratio obtained by using the nTMS-based technique as compared to the standard one for each fascicle. These values were higher than the 75% suggested as optimal by Frey et al. for CST (Frey et al., 2012) for three of four language fascicles. Therefore, the use of such FA ratio values could be considered a good strategy to obtain a reliable and standardized DTI-FT of AF, ILF, IFOF, and UF.

These findings suggests that the nTMS-based DTI-FT, and in particular the “single spot” strategy could improve the visualization of the complex cortico-subcortical language network, either by increasing the identification of cortico-subcortical connections, or by reducing the computation of aberrant fibers. In order to provide a preliminary statistical validation of the nTMS “single spot” approach, we analyzed the presence of an eventual concordance between the presumed function of each language fascicle and the most frequently observed error type among connected spots. We observed a perfect concordance for the IFOF and the ILF, supposed to be involved in the semantic aspect of language (Dick and Tremblay, 2012; Chang et al., 2015). Our analysis showed that both these fascicles were connected to a significantly higher number of spots corresponding to semantic errors as compared to the other error types. A good concordance was observed for the AF that is presumed to be involved both mainly in the articulatory but also in semantic processing (Dick and Tremblay, 2012; Chang et al., 2015). We found a prevalence of hesitation and semantic errors, whereas other error types were significantly fewer. Lastly, we found that nTMS spots connected to the UF were mainly semantic, hesitation and no response errors. However, the different distribution of error types was not significant. Yet, such a distribution can be attributed to the role of the UF in the semantic processing (Dick and Tremblay, 2012; Chang et al., 2015) and in the proper name retrieval process (Papagno, 2011). Therefore, if semantic connections reflect its role in the ventral stream, hesitation and no response errors could be interpreted as an expression of anomia, that has been reported as a consequence of UF resection or damage (Papagno et al., 2011, 2016). These data further increased our confidence in the nTMS-based approach.

Moreover, even though the first step of the nTMS-based approach is the computation of all the main language fascicles through the standard technique, its combination with the objective mapping obtained through repetitive nTMS used as seeding region could reduce the operator-variability that hampers standard DTI-FT, increasing its accuracy also for language tracts. However, that does not necessarily mean that, by using nTMS-positive spots as second seeding ROIs, connections with fascicles are always identified for each single nTMS-positive spot. Fiber connections are visualized only if DTI analysis identified a preferential water diffusion in that specific direction toward the nTMS-positive spot. The visualization of these connections is less accurate by using the standard approach, which is based only on the choice of anatomical landmarks as seeding ROIs.

The reliability of the nTMS-based approach, especially the “single spot” strategy, is further underlined by a significant higher likelihood of subcortical connections with nTMS-positive spots as compared to nTMS-negative ones. Indeed, true-positive connections are visualized up to 4-fold more frequently than false-positive ones. Hypothetically, the larger the difference between the likelihood of true-positive and false-positive connections, the higher is the reliability of the nTMS-based reconstruction of the language network.

The potential clinical role of the present technique could be similar to that reported for the CST in previous studies (Conti et al., 2014; Frey et al., 2014). In particular, Frey et al demonstrated that the nTMS-based DTI-FT of the CST is able to improve surgical outcome of patients operated on for motor-eloquent tumors. Thanks to functional information provided by nTMS mapping, the present technique, especially the “single spot” strategy, could result in a more accurate reconstruction of the complex cortico-subcortical language network. This could increase surgeon's confidence with the language network, improve the preoperative planning and guide intraoperative monitoring and tumor resection, leading to a safer surgical management of brain tumors located near language cortical and subcortical structures. Nevertheless, the validation of these hypotheses goes beyond the aim of the present study. Further studies are warranted in this direction.

Yet, the present study has some limitations. It has been performed on a limited number of patients and our findings must to be confirmed by comparison to the gold standard IONM in order to definitively verify the accuracy of the technique. Moreover, this new approach for an nTMS-based DTI-FT of language tracts shares the same limitation of nTMS speech mapping, especially a reduced accuracy in case of patients with evident preoperative language deficits.

Another limitation of the nTMS language mapping is that its positive predictive value has been reported to be very variable, ranging from 35.6 to 69% (Picht et al., 2013; Tarapore et al., 2013). However, latest reports suggest that it can be increased by further refinement of the existing methodologies (Krieg et al., 2014). This can improve the accuracy of the technique and the reliability of the language positive spots detected by nTMS.

Another criticism could be the use of a DTI protocol with 20 directions that could be considered not sufficient to perform a high resolution fiber mapping. As reported in the literature, the minimum number of directions required to compute DTI is six (Basser et al., 1994a,b). Several papers reported that increasing the total number of directions could increase DTI-FT accuracy improving the estimation of some scalar parameters such as fractional anisotropy at the expenses of a longer scanning time (Lebel et al., 2012; Yao et al., 2015). Nevertheless, increasing the number of fibers does not necessarily improve visualization of the main white matter fascicles (Lebel et al., 2012). Moreover, it has been reported that a total number of direction between 18 and 21 provides a robust estimation of diffusion anisotropy and can improve the repeatability/reliability of DTI experiments (Papadakis et al., 2000; Zhan et al., 2010). In the clinical settings, a protocol with 20 directions can be considered a good compromise between DTI accuracy and the scanning time.

Finally, the nTMS-based DTI-FT approach we described in the present paper should be best considered as a preliminary exploration of “one” possible way of defining language-tract-connected spots, and not necessarily the best one. Other nTMS-based technical strategies might lead to different specificity, sensitivity, and accuracy as compared to our approach. In particular, DTI methodology applying probabilistic algorithms might further improve the nTMS-based approach by reducing the probability of false-positive fibers due to crossing fibers or false-negative fibers by edema disruption of the eigenvector. Further studies in this direction are warranted.

## Conclusions

nTMS-based DTI-FT of language subcortical tracts is feasible and provides an improved visualization of the cortico-subcortical language network compared to the standard atlas-based DTI-FT. Higher FA values and more successful visualization of cortico-subcortical connections increase the reliability of the nTMS-based approaches in comparison to atlas-based strategies. Furthermore, error-type specific tracking of cortico-subcortical language fibers opens a whole new avenue to a novel, site and function specific analysis of the intrinsic language network surrounding the brain tumor of the individual patient.

## Author contributions

TP designed the study, analyzed and interpreted data, revised the work, approved the final version and confirmed the integrity of the whole work. GR designed the study, retrieved the majority of the data, interpreted the data, drafted the work, approved the final version and confirmed the integrity of the whole work. IB contributed to the design of the work, retrieved and analyzed data, revised the work, approved the final version and confirmed the integrity of the whole work. HS retrieved data, managed the study-database and coordinated patient inclusion, revised the work, approved the final version and confirmed the integrity of the whole work. AG and PV contributed to the design of the study, revised the work, approved the final version and confirmed the integrity of the whole work.

## Funding

TP received funding from the Deutsche Forschungsgemeinschaft (DFG), grant no. EXC1027/1, in conjunction with this study.

### Conflict of interest statement

The authors declare that the research was conducted in the absence of any commercial or financial relationships that could be construed as a potential conflict of interest.

## Abbreviations

ADC: apparent diffusion coefficient
AF: arcuate fascicle
aMTG: anterior middle temporal gyrus
anG: angular gyrus
aSMG: anterior supramarginal gyrus
aSTG: anterior superior temporal gyrus
CST: corticospinal tract
DEC: directionally encoded color
DICOM: digital imaging and communications in medicine
dPoG: dorsal postcentral gyrus
dPrG: dorsal precentral gyrus
DTI: diffusion tensor imaging
DTI-FT: diffusion tensor imaging fiber tracking
FA: fractional anisotropy
FDI: first dorsal interosseus
IFOF: inferior fronto-occipital fascicle
ILF: inferior longitudinal fascicle
IONM: intraoperative neurophysiological monitoring
IPI: inter-picture interval
mMFG: middle middle frontal gyrus
mMTG: middle middle temporal gyrus
mPoG: middle postcentral gyrus
MPR: multi-planar reconstruction
mPrG: middle precentral gyrus
MRI: magnetic resonance imaging
mSFG: middle superior frontal gyrus
mSTG: middle superior temporal gyrus
nTMS: navigated transcranial magnetici stimulation
opIFG: opercular inferior frontal gyrus
pMFG: posterior middle frontal gyrus
pMTG: posterior middle temporal gyrus
pSFG: posterior superior frontal gyrus
pSMG: posterior supramarginal gyrus
pSTG: posterior superior temporal gyrus
RMT: resting motor threshold
ROI: region of interest
SPL: superior parietal lobe
TE: echo time
TI: inversion time
TR: relaxation time
trIFG: triangular inferior frontal gyrus
UF: uncinate fascicle
VAS: visual analogue scale
vPrG: ventral precentral gyrus
vPoG: ventral postcentral gyrus
trIFG: triangular inferior frontal gyrus.
